# Hydrogen Gas Inhalation Regressed Coronary Artery Aneurysm in Kawasaki Disease-Case Report and Article Review

**DOI:** 10.3389/fcvm.2022.895627

**Published:** 2022-05-12

**Authors:** Ho-Chang Kuo

**Affiliations:** ^1^Kawasaki Disease Center, Kaohsiung Chang Gung Memorial Hospital, Kaohsiung, Taiwan; ^2^Department of Pediatrics, Kaohsiung Chang Gung Memorial Hospital, Kaohsiung, Taiwan; ^3^College of Medicine, Chang Gung University, Taoyuan, Taiwan; ^4^Department of Respiratory Therapy, Kaohsiung Chang Gung Memorial Hospital, Kaohsiung, Taiwan; ^5^Taiwan Association for the Promotion of Molecular Hydrogen, Kaohsiung, Taiwan

**Keywords:** Kawasaki disease, hydrogen gas, inhalation, aneurysm, regression

## Abstract

Kawasaki disease (KD) is a systemic vasculitis that primarily affects children under the age of 5 years old and is among the most common acquired heart disease in developed countries, particularly in Asia. No effective treatment is currently available for aneurysm formation in KD. In this report, we showed a KD patient with an aneurysm over the right coronary artery with a size of 6.08 mm in diameter and 35 mm in length, which completely regressed to within normal range after hydrogen inhalation within 4 months after disease onset. This 10-year-old KD patient was diagnosed on the 12th day of disease onset with incomplete presentation of KD symptoms. Intravenous immunoglobulin was prescribed after KD diagnosis was confirmed by the formation of a coronary artery aneurysm. Once discharged from the hospital, the family used hydrogen inhalation (77% hydrogen and 23% oxygen) at home with nasal cannula 1 h per day. The aneurysm was found to be completely regressed at the 4-month follow-up (day 138 of the illness). The follow-up laboratory data showed complete blood cell count, differential count, electrolytes, liver enzyme, and renal function to all be within normal range. This is the first study to report an aneurysm from KD with regression under supplementary therapy with hydrogen gas inhalation and no other complications. Therefore, hydrogen gas inhalation may be an alternative anti-free radical or anti-oxidant therapy for KD, but further study is still required.

## Introduction

Kawasaki disease (KD) is the most common acquired heart disease among children in many countries, especially Asian ones. This acute febrile systemic vasculitis was first reported by Dr. Tomisaku Kawasaki in 1967 in Japanese and 1974 in English (1). Initially referred to as mucocutaneous lymph node syndrome (MCLS), it was later renamed Kawasaki Disease (KD) or Kawasaki syndrome after Dr. Kawasaki (1925–2020) in memory of his contribution. KD mainly affects young children under the age of 5 years old, especially those of Asian descent in Japan, Korea, China, and Taiwan. Currently, the etiology of KD remains unknown (2–4), but both genetic background and environmental impacts have been shown to be important for disease susceptibility.

Aneurysm formation is considered the most severe complication of KD survivors since IVIG treatment was introduced in 1983. According to the American Heart Association (AHA) statement for 2017, a large or giant aneurysm (> 8 mm in diameter) does not “resolve,” “regress,” or “remodel.” Friedman et al. (5) reported a total of 2,860 KD patients and found that 17% had aneurysm; the probability of regression of a moderate size aneurysm was 0.3 at 4 months after disease onset and 0.5 at 24 months. Kato et al. (6) reported outcomes in 598 KD patients and found that aneurysms were diagnosed in 25%, with 49% reducing to a normal luminal dimension 6 to 18 months later.

According to the standard treatment for KD suggested by AHA, no effective anti-inflammatory treatment for aneurysm formation is available after the acute stage. When a KD patient with aneurysm formation after acute stage treatment, antiplatelet or anticoagulation medication is most likely to be prescribed. However, no treatment is available for the inflammation of vasculitis. Nevertheless, considerable evidence has shown ongoing inflammation in the coronary artery of KD. Serological evidence of ongoing systemic inflammation has been noted in those patients with persistent aneurysms, with higher levels of serum amyloid A and interleukin-6, high sensitivity C-reactive protein, hypermethylation of FcγR2B in leukocytes, and imaging evidence by PET scanning (7). Additional anti-inflammation treatment may be needed for KD patients with aneurysm formation. In this study, we reported on a KD patient with regressed moderate aneurysm formation after hydrogen gas inhalation and reviewed the revelant literature.

## Case

**Figure 1:**
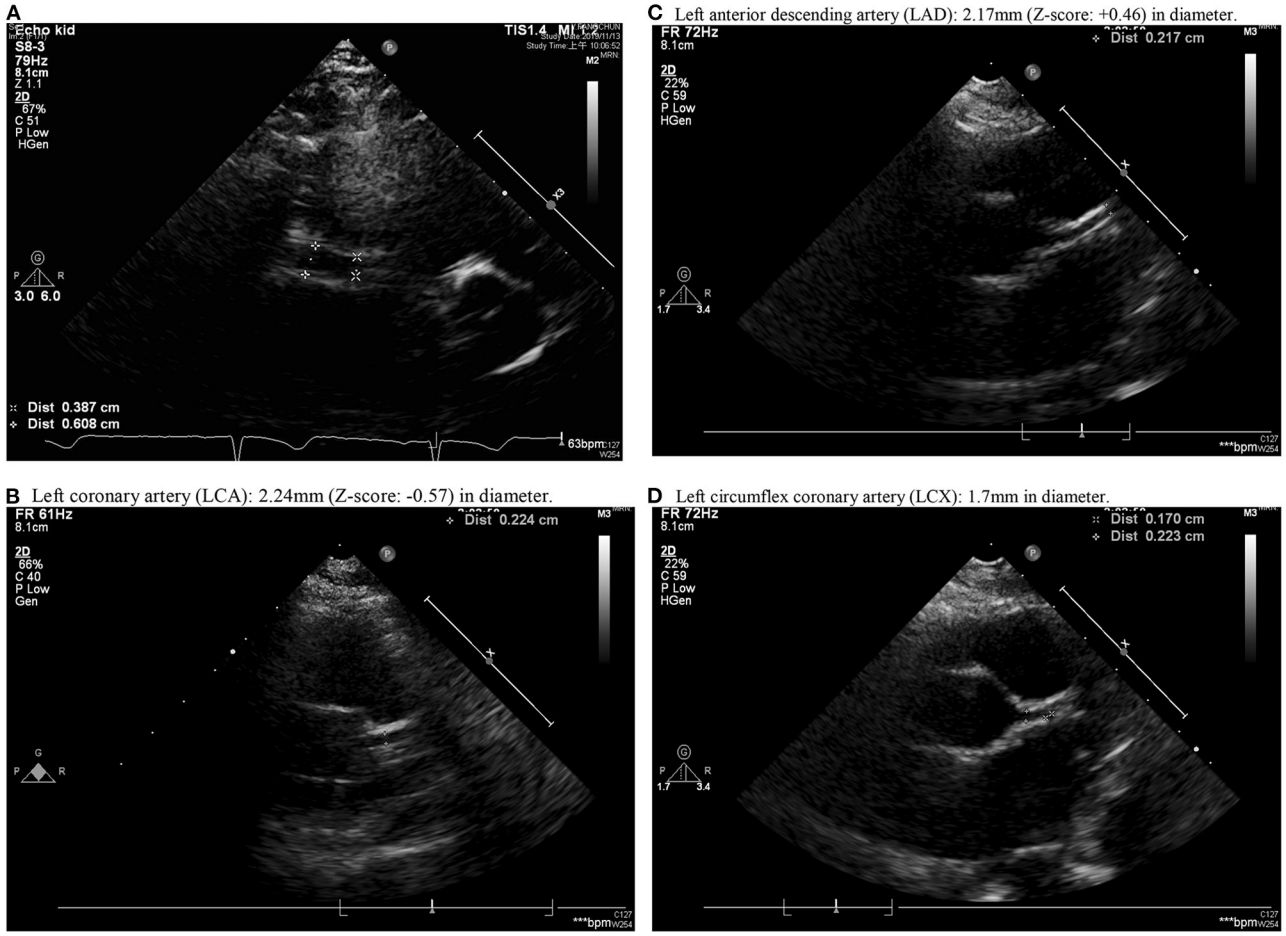
Aneurysm formation over middle right coronary artery (RCA) with a diameter of 2.9 mm near the ostia, gradual dilation to maximum diameter of 6.08 mm near the middle 1/3 of RCA with length of 3.5 cm and distal RCA diameter of 3.11 mm (posterior AV groove) **(A)**. **(B–D)** showed Left coronary artery (LCA), Left anterior descending artery (LAD) and Left circumflex coronary artery (LCX). **(B)** Left coronary artery (LCA): 2.24 mm (Z-score: −0.57) in diameter. **(C)** Left anterior descending artery (LAD): 2.17 mm (Z-score: +0.46) in diameter. **(D)** Left circumflex coronary artery (LCX): 1.7 mm in diameter.

**Figure 2:**
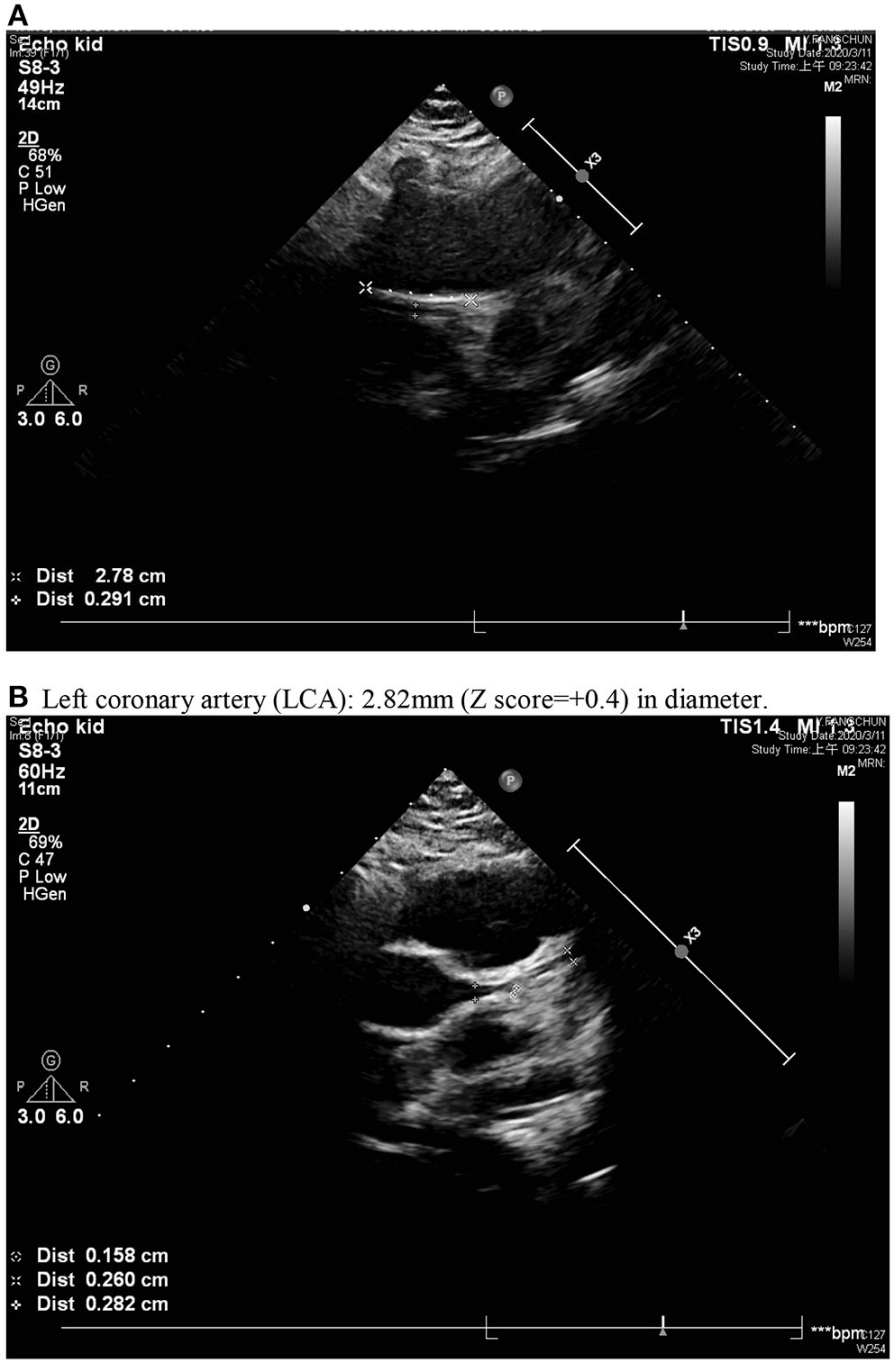
Normal inner diameter and origin of coronary arteries; regressed right coronary artery with a diameter of 2.91 mm **(A)** and normal left coronary artery (LCA) **(B)**. **(B)** Left coronary artery (LCA): 2.82 mm (Z score = +0.4) in diameter.

A previously healthy 10-year-old Taiwanese boy presenting with a high fever for 12 days was admitted to another hospital in year 2019. Prior to admission to our hospital, he showed bilateral conjunctival injection, erythema of the lips and neck, and lymphadenopathy. No BCG site induration was observed, nor was skin rash or limbs induration found. After the patient was admitted to our pediatric ward, the laboratory analysis revealed evidence of mild leukocytosis (10,600/μl), thrombocytosis (557,000/μl), and high C-reactive protein (CRP) (132.22 mg/L), without acute liver or kidney injury. A two-dimensional echocardiography on day 1 of admission (day 12 of illness) revealed a 4.12 mm aneurysm (BSA-adjusted Z score = 3.17) of the right coronary artery (RCA) with general dilatation of the RCA and LCA. High-dose intravenous immunoglobulin (IVIG, 2 g/kg) infusion was prescribed during admission (day 12 of illness). After being discharged, the RCA still progressed to a mid-sized aneurysm 6.08 mm (Z score: 4.85) in diameter and 35 mm in length (day 20 of illness) (Figure 1). Parents were directed to give hydrogen gas inhalation at least 1 h per day for the patient (77% hydrogen with 23% oxygen, 70~75 liter/hour) at home by themselves until aneurysm regression. The follow-up echocardiography showed regression in the aneurysm, which was then 5.37 mm in diameter and 12 mm in length of RCA (day 34 of illness) and 4.56 mm × 8.68 mm (day 48 of illness), then 4.16 mm (day 62 of illness). The mid-sized aneurysm regressed to within normal range with 2.91 mm in diameter (Z score = 1.46) on day 138 of illness (Figure 2). The following laboratory data showed normal liver enzyme (aspartate aminotransferase/ alanine aminotransferase: 24/17 U/L), renal function (blood urine nitrogen/creatinine: 18.0/0.52 mg/dl, estimated glomerular filtration rate: >60 ml/min), total white blood cell count: 11,800/ul (leukocytosis), hemoglobin: 14.2 g/dl, platelet: 258,000/ul, segment: 67%, lymphocyte: 26%, monocyte: 5%, eosinophil: 0%, basophil: 0%, sodium: 143 mEq/L, potassium: 3.7 mEq/L, chloride: 107mEq/L, albumin: 4.6 g/dl, calcium: 9.7 mg/dl, eosinophil cationic protein: <2.0 microgram/L and total immunoglobulin E: 107 KU/L. The laboratory data from acute and chronic stage (after hydrogen gas inhalation) were showed in Table 1. We reported on this case using a medical chart review retrospective, and the institutional review board (IRB) of Chang Gung Memorial Hospital approved this study (IRB No.: 201900827B0).

**Table 1:** Laboratory data from acute stage and chronic stage (after hydrogen gas inhalation) of Kawasaki disease.

**Laboratory data**	**First day of admission (day 12 of illness)**	**Followed-up (day 138 of illness)**
White blood cell count (/ul)	10,600	11,800^*^
Hemoglobin (g/dl)	11.6^*^	14.2
Platelet (/ul)	557,000^*^	258,000
Segment (%)	67.2	67
Lymphocyte (%)	22.5	26
Monocyte (%)	8.2	5
Eosinophil (%)	1.6	0
Basophil (%)	0.5	0
Aspartate aminotransferase (U/L)	25	24
Alanine aminotransferase (U/L)	27	17
Blood urine nitrogen (BUN) (mg/dl)	10.0	18.0
Blood creatinine (mg/dl)	0.63	0.52
Estimated glomerular filtration rate (ml/min)	>60	>60
Sodium (mEq/L)	139	143
Potassium (mEq/L)	3.9	3.7
Chloride (mEq/L)	102	107
Albumin (g/dl)	3.69	4.6
C-reactive protein (mg/L)	132.22^*^	<5

* *indicate data not within normal range*.

## Discussion and Review

Coronary artery aneurysm formation is the most severe complication of KD and may have life-long implications. Currently, no effective treatment is available for KD patients with aneurysm formation after the acute stage of IVIG treatment, and it is even worse when the patient has a progressed aneurysm. Only antiplatelet and anticoagulant medications are prescribed for KD patients with aneurysm formation, and these agents had no effect on anti-inflammation for coronary vasculitis. Additional anti-inflammatory agents, including steroids, tumor necrosis factor blocker, and immunomodulatory agent have been suggested for KD patients with IVIG resistance in the acute stage during admission at the hospital. However, these anti-inflammatory agents have not been prescribed for KD patients with an already formed coronary aneurysm.

Wang et al. reported that nitric oxide (NO)-mediated inflammatory responses play a very important role in the pathogenesis of coronary artery lesions of KD (8). Yahata et al. (9) reported that inflammation and oxidative stress are closely related to a variety of diseases. Oxidative stress plays an important role in the pathology of KD and even multi-systemic inflammatory syndrome (MIS-C) (10, 11). The excessive production of reactive oxygen species (ROS) increases oxidative stress, which triggers an endless and vicious cycle of inflammation reactions and ROS metabolites. Oxidative stress has been shown to have an important role in the development of arteriosclerosis of KD patients and is strongly associated with endothelial dysfunction in early childhood patients with KD (12). Furthermore, simultaneous oxidative and nitrative stress occurrences in KD patients may lead to cardiovascular complications (13). IVIG treatment in KD can effectively reduce oxidative stress that provokes vasculitis. The urinary level of 8-iso-prostaglandin F2alpha (8-iso-PG) is a useful marker of the effectiveness of IVIG on oxidative stress of KD (14). The risk factors for the development of atherosclerosis in adults, such as CRP, oxidative stress, and inflammatory cytokines are also increased in both the remote phase of KD and the acute stage (15). Taken altogether, oxidative stress and nitrative stress play important roles in the pathogenesis of vasculitis and coronary artery lesions of KD.

Ohsawa et al. (16) reported that hydrogen gas, an inert gas, is an effective antioxidant that contributes to the regulation of oxidative stress and inflammation response. Their study demonstrated that H2 reduced oxidants of the detrimental ROS, thus protecting against oxidant-induced cell injury. Hydrogen gas inhalation has been reported to decrease the levels of inflammatory cytokines including TNF-α, IL-1β, IL-6, and hypoxia-inducible factor 1 alpha (17), which were found to be elevated in KD and even higher in KD with CAL formation. Long et al. reported that hydrogen gas had protective effects in a rat model of branch retinal vein occlusion via decreasing Vascular endothelial growth factor (VEGF)-α expression (18). Local VEGF-α and its signaling pathway are associated with the development of LCWE-induced CAL in mice model of KD (19). Zhang et al. also reported that post-conditioning with hydrogen gas ameliorated subarachnoid hemorrhage (SAH)-induced, which is predominantly caused by a ruptured aneurysm, neuronal pyroptosis in part through the mitochondrial ATP-sensitive K+ channels/ERK1/2/p38 MAPK signaling pathway (20). These evidences support that hydrogen gas may be effective on rupture or occlusion of vasculitis associated with KD. Cole et al. showed that inhalation of hydrogen gas does not appear to cause clinically significant adverse effects in healthy adults. Although these data suggest that inhaled hydrogen gas may be well tolerated, future studies need to be powered to further evaluate safety especial in children (21).

Altogether, oxidative stress and inflammatory cytokines that were elevated in KD and CAL formation may benefit from hydrogen gas inhalation. The correlation between aneurysm regression and suppression of inflammatory cytokines after hydrogen gas inhalation in KD also need future investigation. After performing the literature review, we determined that we retrospectively reported the first case of a KD patient with coronary aneurysm formation to regress after hydrogen gas inhalation.

## Data Availability Statement

The original contributions presented in the study are included in the article/supplementary materials, further inquiries can be directed to the corresponding author/s.

## Ethics Statement

The studies involving human participants were reviewed and approved by Chang Gung Memorial Hospital. Written informed consent to participate in this study was provided by the participants' legal guardian/next of kin. Written informed consent was obtained from the minor(s)' legal guardian/next of kin for the publication of any potentially identifiable images or data included in this article.

## Author Contributions

The author confirms being the sole contributor of this work and has approved it for publication.

## Conflict of Interest

The author declares that the research was conducted in the absence of any commercial or financial relationships that could be construed as a potential conflict of interest.

## Publisher's Note

All claims expressed in this article are solely those of the authors and do not necessarily represent those of their affiliated organizations, or those of the publisher, the editors and the reviewers. Any product that may be evaluated in this article, or claim that may be made by its manufacturer, is not guaranteed or endorsed by the publisher.
